# Opioid prescription patterns among patients who doctor shop; Implications for providers

**DOI:** 10.1371/journal.pone.0232533

**Published:** 2020-05-26

**Authors:** Todd Schneberk, Brian Raffetto, Joseph Friedman, Andrew Wilson, David Kim, David L. Schriger

**Affiliations:** 1 Department of Emergency Medicine, Los Angeles County +University of Southern California, Los Angeles, CA, United States of America; 2 David Geffen School of Medicine at University of California, Los Angeles, CA, United States of America; 3 Department of Neurology, Greater Los Angeles VA, Los Angeles, CA, United States of America; 4 Department of Neurology, University of California, Los Angeles, CA, United States of America; 5 Department of Emergency Medicine, University of California, Los Angeles, CA, United States of America; University of South Australia, AUSTRALIA

## Abstract

**Introduction:**

Patients who doctor shop for opioids are a vulnerable population that present a difficult dilemma for their health care providers regarding best methods of immediate treatment and how to manage their risk of harm from opioids. We aim to describe and compare opioid prescription patterns among high quantity prescription patients who doctor shopped, high quantity prescription patients who did not (doctor shopping eligible patients), and the remaining patients who received opioid prescriptions to guide population health policies for high risk opioid use patients.

**Methods:**

We performed a cross-sectional descriptive analysis of opioid prescriptions during an 8-year period using California’s de-identified Controlled Substance Utilization Review and Evaluation System (CURES) database from years 2008–2015. We identified the prevalence of patients who doctor shopped and depicted their opioid prescription patterns including prescriber characteristics, in comparison to the aforementioned groups. Doctor shopping was defined by patients who received greater than 6 or more prescriptions from at least 6 different prescribers within 6 months of time.

**Results:**

Among the 3 million individuals who received an opioid prescription during the 8-year period, 1.3% met the doctor shopper definition. These patients received high levels of chronic opioids with 82% and 33% averaging greater than 20 and 100 morphine milligram equivalents (MME) daily, respectively, in comparison to 72% and 18% in the doctor shopping eligible group. Patients who doctor shopped received a significant proportion of their MME from 1 main prescriber (54%) and only received 2–5% of their total MME from episodic care providers, despite 88% receiving a prescription from these providers.

**Conclusions:**

Patients who doctor shop are at high risk of opioid use disorder but represent a small fraction of those with dangerous opioid use. Furthermore, these individuals do not receive substantial opioids from episodic providers, which challenges the utility of prescription reduction programs in curbing use among this population. These results suggest we re-evaluate physician roles in the care of these patients and focus on referral to treatment and harm reduction strategies.

## Introduction

Prescriptions for opioids nearly quadrupled from 1999–2008, and overdoses followed a similar pattern, resulting in state efforts to identify individuals most at risk for opioid abuse and overdose [1–3]. The act of “doctor shopping”–receiving opioid prescriptions from multiple providers–was identified as a risk factor for opioid use disorder, overdose, diversion, and higher health risks [4, 5]. As a result, states developed Prescription Drug Monitoring Program (PDMP) databases in part, to flag patients who doctor shop [6]. Recommendations for the healthcare providers who find themselves caring for a patient who is doctor shopping are not clearly defined, and there is potential to increase stigmatization [7]. In addition, paradoxically, there is the potential to produce negative health outcomes as denied patients can pursue opioids through riskier channels or use more lethal forms of opioids [8]. At present, there is evidence that PDMPs may reduce overall opioid prescriptions depending on how they are operationalized, but there is insufficient evidence to determine if PDMPs curtail doctor-shopping behavior, or reduce negative patient centered outcomes, such as opioid overdoses [9–11].

The scale of the opioid crisis demands well informed, targeted efforts to reduce harm and find opportunities for intervention along the spectrum of opioid use. For this reason, we analyzed the California PDMP database from 2008 to 2015 to describe the opioid prescription pattern[s] of individuals who met criteria for doctor shopping and to compare them to those of individuals who did not meet doctor-shopping criteria. Our goal was to use these empiric data to contextualize the discussion around doctor-shopping behavior and to inform future initiatives to address the opioid epidemic.

## Methods

Details about our acquisition and processing of the California’s Controlled Substance Utilization Review and Evaluation System (CURES) database can be found in our prior paper [12]. In brief, we utilized data obtained from CURES—part of the Prescription Drug Monitoring Program (PDMP)–during 2008 to 2015. Using Stata 14.2 (Stata Corp., College Station TX), we developed the following measures for each patient in the database: time between first and last prescription, time receiving opioid medications, mean and median morphine milligram equivalents (MME) per day, mean and median MME per day for the 90-day period of maximal use, and doctor shopping status (defined below). We also identified each patient’s primary opioid prescriber and the category of provider who wrote each prescription. Options for this category included: primary provider, 1-prescription provider (a provider who wrote a single prescription for the patient in the entire 8-year database), 2-prescription provider, and other. Operational definitions of each variable can be found in S1 Appendix.

We based our definition of doctor shopping behavior on California’s PDMP’s Patient Safety Alert criteria: any person who obtains prescriptions from 6 or more prescribers or 6 or more pharmacies during a 6-month period [13]. For this paper we included only those patients who met the provider definition (not multiple pharmacies) as this was our group of interest. We defined a “doctor shopping eligible” comparison group as those patients who could have doctor shopped (they had at least one 183-day period when they received 6 or more prescriptions) but never met the doctor shopping definition. That is to say, during that 183-day period, their 6 or greater prescriptions were not from 6 or more providers, indicating that their opioid prescription source was more uniform. A third category was defined as those who did not meet either definition, and by default had much less prescription opioid use. These categories allowed for comparison of doctor shoppers with those who had similar opioid utilization with a more constant prescription source. It also separated these high use groups from the lower utilization patients who are less likely to be analogous to those who doctor shopped.

In addition to describing mean and median usage we also categorically grouped patients into those who consumed greater than 20 MME/day and those who consumed great than 100 MME/day, overall and during each patient’s 90-day period of maximal use. These categories of use were associated with higher risk of overdose in prior literature [14–16].

We used STATA 14.2 (Stata Corp., College Station, TX) to create graphical depictions of cross-tabulations of doctor shopping status and the relevant independent variables. Our purpose is descriptive. With such a large N, we could use formal testing to establish that a host of comparisons are significant. Supported by an increasing literature demonstrating the problems with statistical testing in observation studies, we opted to describe our findings–with appropriate measures of variance–rather than test them [17–19]. The study was considered exempt by the UCLA Institutional Review Board.

## Results

The 10% random sample from the CURES dataset included 17,954,968 opioid prescriptions written to 3,044,579 patients by 185,424 prescribers. Most prescriptions (87.7%) were for short-acting opioids; hydrocodone (62%) and oxycodone (10.4%) predominated (see S2 Appendix).

### Prevalence of different patient categories based on prescription patterns

**Fig 1:**
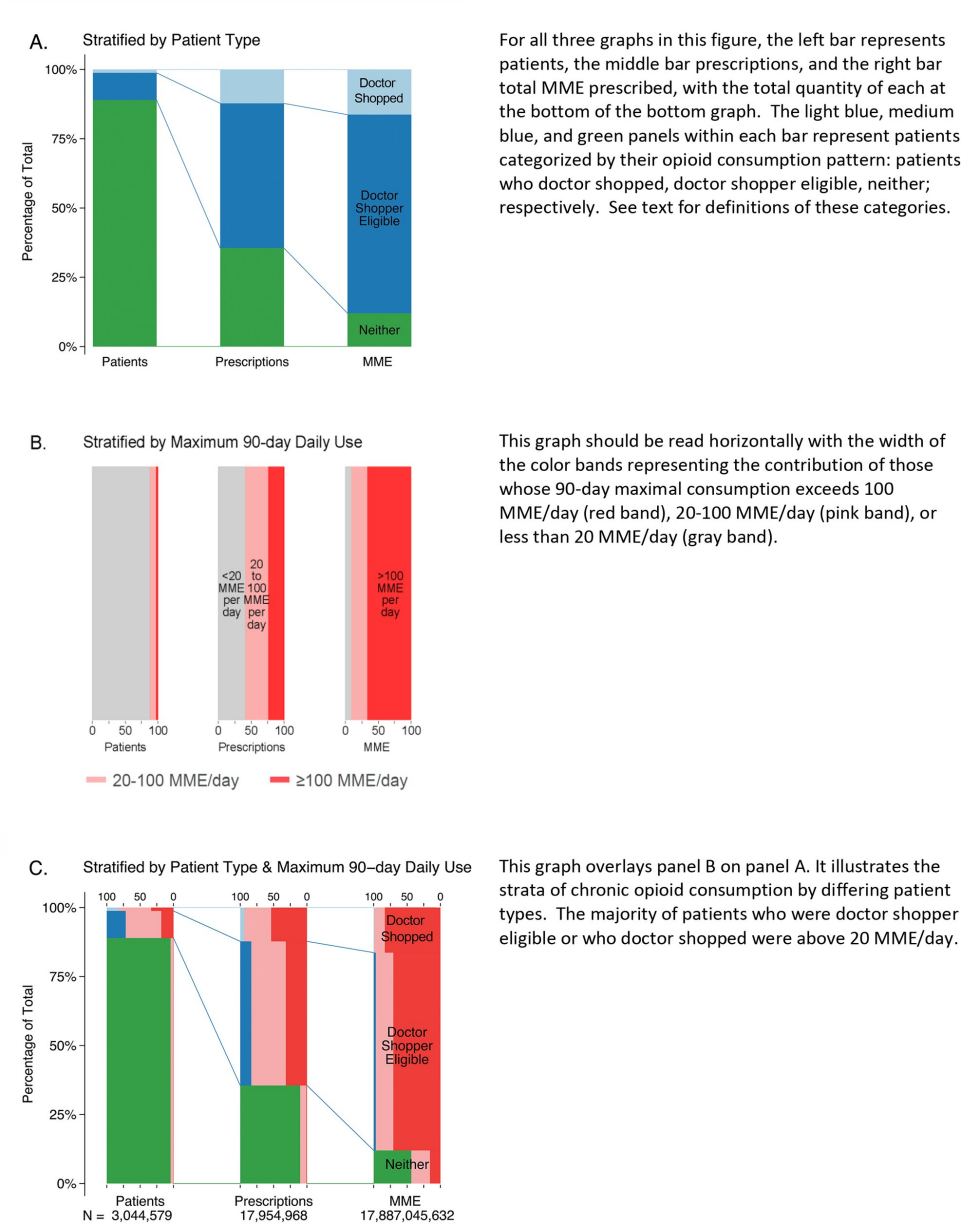
Patients, prescriptions, and Morphine Milligram Equivalent (MME) prescribed, by patients & prescriber characteristics.

Of the ≈3 million individuals getting at least 1 opioid prescription during the 8-year study period, 37,333 patients (1.3%) met the doctor shopping definition at some time. Another 298,493 doctor shopping eligible patients (9.7%) received sufficient prescriptions—at least 6 in a 6-month period—to be doctor shopping but did not doctor shop. The remaining 2.7 million patients (89%) did not meet either definition (the “Neither” group). (Fig 1, left bar)

### Opioid prescription patterns among patients who doctor shop

**Fig 2:**
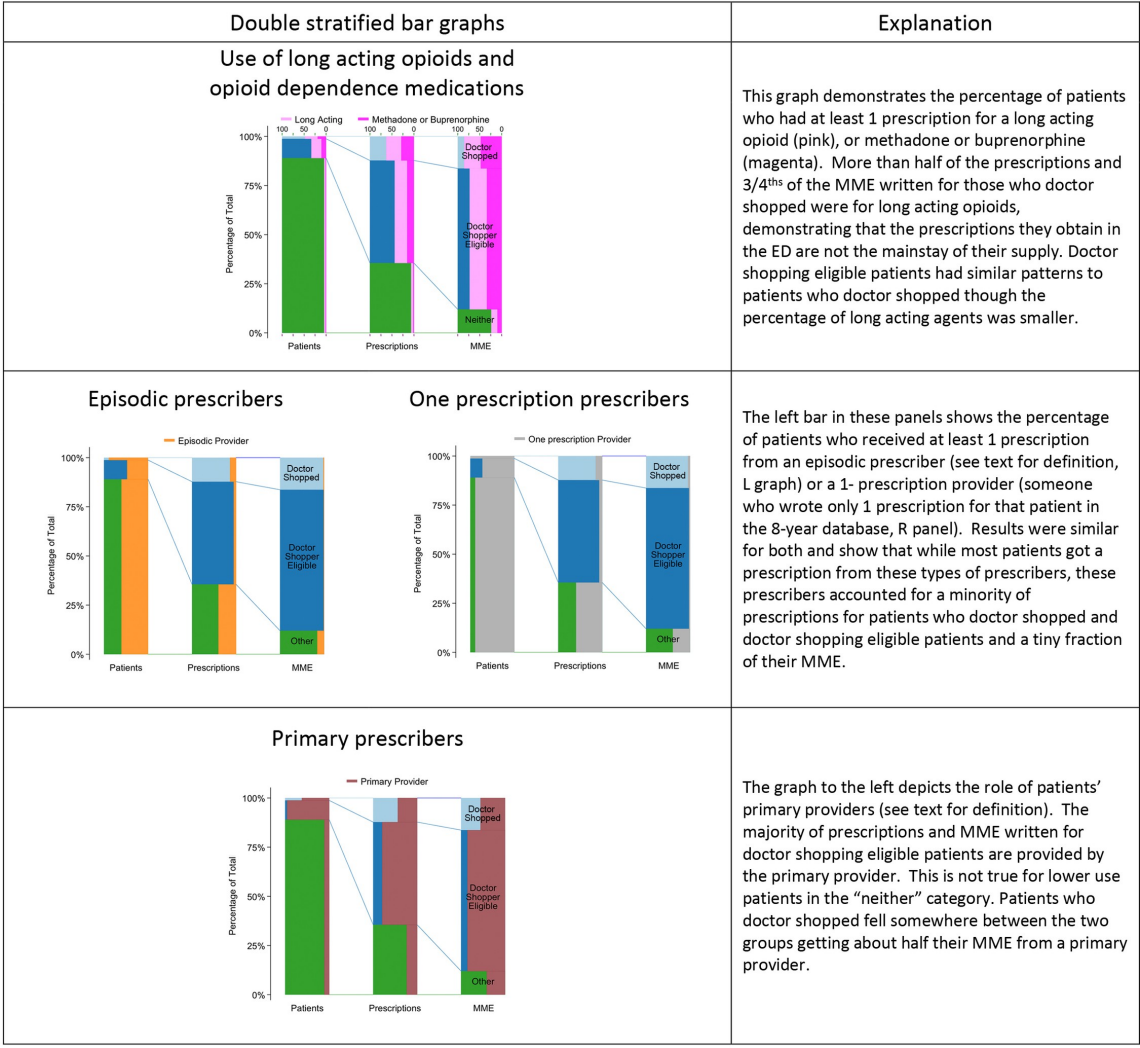


Over the 8-year data period, doctor shopping patients received 59 prescriptions during a 4.3-year span (1,563 days) between their first and last prescription (Table 1). They had opioid medication coverage for a mean of 1,216 days (81%) during that span (See S1 Appendix for methods and definitions). Their prescriptions came from a mean 14.7 prescribers. Median daily consumption on days when they had prescriptions for medications was 22.9 MME [IQR 9.9, 53.1] and daily median consumption during the 90-day period of maximum consumption was 59.6 MME [IQR 26.7, 138.5]. 82.1% of patients who doctor shopped had a maximum daily 90-day MME greater than 20; 33.4% exceeded 100 MME/day (Fig 2A). Doctor shopping patients received 83.7% short acting opioid prescriptions; 51.9% received only short acting opioids. Hydrocodone/acetaminophen was the most common (56.6%), followed by oxycodone/acetaminophen (9.1%) and morphine sulfate (6%). 18.9% of patients who doctor shopped received at least 1 prescription for buprenorphine or methadone (Fig 1E). These medications accounted for 32.6% of all long acting prescriptions and 5.4% of all prescriptions written for this group (Fig 1E).

**Table 1:** Patient characteristics (mean, median [IQR] unless otherwise indicated).

	Doctor Shopping Patients		Doctor Shopping Eligible Patients		Neither	
**Patients, N (%)**	**37,333 (1.3%)**		**298,493 (9.7%)**		**2,708,757 (89%)**	
Demographics						
Age	50	51 [40, 59]	56.9	57 [47, 67]	49.2	49 [33, 64]
Female (%)	59.8%		57.3%		56.8%	
Time data						
Total span (days)	1563	1586 [731, 2376]	1262	1084 [431, 2055]	427	90 [90,484]
Active time (days)	1216	1035 [523, 1811]	921	667 [383, 1302]	170	90 [90, 180]
Percent active	80.9% 89.9% [67%, 100%]		80.2% 92.7% [63.5%, 100%]		46.3% 39.5% [27.7%,60.6%]^a^	
Prescription data						
Prescriptions/patient	58.9	39 [19,80]	31.4	19 [11, 39]	2.4	1 [1, 3]
Prescriptions/patient/month	1.39	1.22 [.92, 1.67]	1.06	.89 [.72, 1.2]	.40	.33 [.33, .41]
MME/prescription (patient weighted)	954	545 [283, 1047]	1043	529 [294, 1025]	270	150 [100, 243]
Prescriber data						
Prescribers/patient	14.7	12 [9, 17]	4.6	4 [2, 6]	1.7	1 [1, 2]
Prescribers/patient/month	.48	.42 [.27, .61]	.21	.17 [.10, .28]	.33	.33 [.33, .33]
Prescriptions/provider (patient weighted)	4.0	2.9 [1.8, 5.1]	8.8	5.7 [3.2, 10]	1.3	1 [1.0, 1.2]
Usage data						
Total morphine equivalents/patient	78190	22070 [6887, 70702]	42969	11025 [4375 32650]	791	225 [120, 600]
Morphine equivalents/patient/day	51.6	22.9 [9.9, 53.1]	40.9	16.5 [8.3, 35.9]	3.9	1.7 [1.1, 3.3]
Maximum 90-day daily MME	133	59.6 [26.7, 139]	81.8	34.5 [17.9, 72.4]	5.4	2 [11, 47]
Percent maximum 90-day daily MME > 20	82.1%		71.7%		4.3%	
Pharmacologic Data: (%)						
Patient gets only short acting opioids	51.9%		66.7%		95.8%	
Patient gets ≥ 1 methadone/buprenorphine	18.9%		10.2%		1%	
**Prescriptions, N (%)**	**2,198,631 (12.3%)**		**9,378,221 (52.1%)**		**6,378,116 (35.5%)**	
Morphine equivalents/prescription	1328	600 [225, 1200]	1368	600 [300, 1200]	336	150 [100, 300]
Short acting opioids (%)	83.7%		84.3%		97.0%	
Methadone/buprenorphine (%)	5.4%		5.3%		.8%	
Top 3 medications (%)	Hydrocodone/Acetaminophen (56.6%)		Hydrocodone/Acetaminophen (59.1%)		Hydrocodone/Acetaminophen (65.3%)	
	Oxycodone/ Acetaminophen (9.1%)		Oxycodone/ Acetaminophen (6.7%)		Codeine/Acetaminophen (11.4%)	
	Morphine sulfate (6.0%)		Morphine sulfate (5.3%)		Oxycodone/ Acetaminophen (6.8%)	

### Comparison of opioid medication patterns between active and inactive doctor-shopping periods

Of the 37,333 patients who met the doctor shopping definition, 3,571 (9.6%) met the doctor shopping definition for their entire time in the database (Table 2). The average doctor shopping patient had a mean 395 days that met the doctor shopping definition and 907 days that did not, doctor shopping for roughly 1/3 of their total time (1,216 days) in the database. These patients received a median 26.9 [IQR 12, 60] MME/day during shopping periods and 17.2 [6, 46] MME/day at other times. Despite median MME per prescription being lower (450 v. 675) during periods of doctor shopping, the increased number of prescriptions per patient per month (median 1.6 v. 0.9) resulted in the increase in daily MME. Short acting opioids comprised 86.1% of prescriptions during doctor shopping periods and 81.9% at other times. The percentage of patients receiving buprenorphine or methadone was similar during doctor shopping and non-doctor shopping periods (13.7% v. 14.2%). Taken together, these data suggest that prescription and MME consumption patterns differ slightly during patients’ doctor shopping and non-doctor shopping periods but both exhibit patterns consistent with chronic opioid use.

**Table 2:** Characteristics of doctor shopping patients during doctor shopping and non-doctor shopping periods (mean, median [IQR] unless otherwise indicated).

	When Doctor Shopping		When not Doctor Shopping	
**Patients, N (%)**	**37,333**		33,762*	
Time data				
Active time (days)	395	288 [228, 437]	907	729 [307, 1390]
Prescription data				
Prescriptions/patient	26.2	16 [10, 28]	36.2	21 [7, 53]
Prescriptions/patient/month	1.8	1.6 [1.2, 2.2]	1.0	.9 [.6, 1.3]
MME/prescription (patient weighted)	837	487 [251, 929]	1067	552 [252, 1142]
Prescriber data				
Prescribers/patient	10.4	7 [6, 11]	6.9	6 [3, 10]
Prescribers/patient/month	.83	.80 [.69, .92]	.30	.28 [.18, .37]
Prescriptions/provider (patient weighted)	2.3	1.9 [1.4, 2.8]	5.0	3.3 [1.7, 6.4]
Usage data				
Total morphine equivalents/patient	27312	8425 [3260, 23330]	56260	12089 [2350, 48028]
Morphine equivalents/patient/day	57.7	26.9 [11.6, 60.2]	46.5	17.2 [5.7, 45.5]
Pharmacologic Data:				
Patient gets only short acting opioids	60.2%		64.2%	
Patient gets ≥ 1 methadone/buprenorphine	13.7%		14.2%	
**Prescriptions, N (%)**	**977,245 (44.5%)**		**1,221,386 (55.5%)**	
Morphine equivalents/prescription	1043	450 [150, 1000]	1555	675 [300, 1500]
Short acting opioids (%)	86.1%		81.9%	
Methadone/buprenorphine (%)	4.3%		6.3%	
Top 3 medications (%)	Hydrocodone/Acetaminophen (57.5%)		Hydrocodone/Acetaminophen (55.8%)	
	Oxycodone/ Acetaminophen (10.5%)		Oxycodone/ Acetaminophen (8.1%)	
	Morphine sulfate (5.4%)		Morphine sulfate (6.4%)	

* 3,571 patients doctor shopped continuously during their time in the data base.

**Fig 3:**
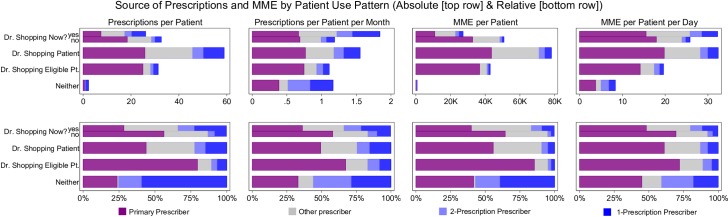
The above figure depicts absolute (top row) and relative (bottom row) distributions of prescriptions per patient, prescriptions per patient per time (months), MME per patient, and MME per patient per time (days), divided into opioids provided by primary prescribers (purple), 1 prescription prescribers (dark blue), 2 prescription prescribers (light blue) and other prescribers (gray) for the different categories of patients. The top row in each individual horizontal bar graph shows patients who doctor shopped divided into distributions when they met the definition of doctor shopping (yes) and when they did not meet the definition (no). The second row in each horizontal bar graph shows the distributions for patients who doctor shopped as the aggregate of all prescriptions during doctor shopping or not. The third row depicts the distributions of opioid prescriptions for those who were doctor shopper eligible and the fourth row is those who fit neither definition.

Prescription patterns differed slightly based on prescriber type during doctor shopping and non-doctor shopping periods. When not doctor shopping, these patients received 8% of prescriptions and 3% of their total MME from one-time prescribers and 56% of their prescriptions and 64% of their total MME from their primary prescriber. When doctor shopping, they received 23% of prescriptions and 9% of their total MME from one-time prescribers and 28% of their prescriptions and 40% of their total MME from their primary provider. These proportions are illustrated in the split horizontal bar in Fig 3, which demonstrates that even when doctor shopping, patients receive a minority of the prescriptions (and MME) from their low frequency prescribers yet maintain a substantial proportion from primary prescribers.

### Comparison of demographic and opioid prescription patterns across categories of patients

Doctor shopping patients were more often female (59.8%) than doctor shopping eligible (57.3%) and other patients (56.8%) (Table 1). The doctor shopping patients (median 51 years, IQR [40, 59]) were younger than doctor shopping eligible patients (57 [47, 67]) and slightly older than other patients (49 [33, 64]) (Table 1).

The 89% of patients who neither doctor shopped nor were doctor shopping eligible used opioids quite differently than these two groups (Table 1, Fig 1A). Over the 8-year course of the database, these individuals averaged 2.4 prescriptions, which they received from 1.7 providers. They averaged 3.9 MME per day when they had a prescription and their maximum 90-day daily MME was 5.4 MME/day. 4.3% of these patients had a maximum 90-day MME that was over 20 MME/day. Only 4.2% ever received a prescription for a long acting opioid and only 1% a prescription for buprenorphine or methadone.

The 9.7% of patients who were doctor shopping eligible and the 1.3% who doctor shopped showed generally similar patterns that were distinct from patients described in the prior paragraph. Compared to doctor shopping eligible patients, patients who doctor shopped had more active days in the database (median 1035 v. 667) and received more prescriptions (mean 58.9 v. 31.4) and more prescriptions per month (median 1.2 v. 0.9). They also had more prescribers (mean 14.7 v. 4.6) and more prescribers per month (median 0.42 v. 0.17). While doctor shopping patients received higher lifetime MME (median 22,070 v. 11,025) than doctor shopping eligible patients, this was largely due to their longer active time in the database, as their median MME/prescription was not appreciably higher (median 545 v. 529).

Slightly over 48% of doctor shopping patients received at least 1 prescription for a long acting opioid compared to 33.3% of doctor shopping eligible patients. A similar pattern was seen for opioids used to treat opioid use disorder (methadone, buprenorphine); 18.9% of patients who doctor shopped and 10.2% of doctor shopping eligible patients received at least one of these prescriptions.

The percentage of patients whose maximum 90-day MME exceeded 20 MME/day was higher for doctor shopping patients than doctor shopping eligible patients (82.1 v. 71.7%) as was the percentage who exceeded 100 MME/day (33.4% v. 18.2% (Fig 1B). The same comparison, using each patient’s mean daily MME over his/her time in the database reveals similar results (54.4% v. 43.2% for ≥ 20MME/day; 12.6% v. 9.0% for ≥ 100 MME/day).

The source of prescriptions was also similar between doctor shopping and doctor shopping eligible patients. Both patient populations received substantial quantities from their primary prescribers (prescriptions 44% v. 80%; MME 56% v. 86%, respectively), and much smaller percentages from the 1-prescription prescribers (prescriptions 15% v. 7%; MME 5% v. 3%, respectively)) (Fig 2). The main difference in prescription supply was from differential contribution from the “other” category. Fig 3 demonstrates the more unstable prescription supply of doctor shopping patients but also illustrates that when not doctor shopping, doctor shopping prescription patterns resemble those of doctor shopping eligible patients.

### Comparison of prescriber characteristics across doctor shopping patient categories

We describe physician prescribing behavior in Table 3. Providers who only wrote prescriptions for doctor shopping eligible and doctor shopping patients wrote prescriptions for fewer patients in our sample (mean 12 and 6 respectively) than providers who wrote for at least one “neither” patient (mean 27). Though they wrote for fewer patients, these providers, wrote more prescriptions per patient (mean 6.7 v. 4.2 v. 1.3, respectively) and the prescriptions were for larger quantities of opioids (311 v. 300 v 189 median MME/prescription, respectively) and larger quantities of opioids per patient (680 v. 567 v. 228 median MME/Patient/Prescriber).

**Table 3:** Prescriber characteristics, N = 185,424 (mean, median [IQR] unless otherwise indicated).

	Doctor Shopping Patients		Doctor Shopping Eligible Patients		Neither	
Prescribers (N, %)	89,683 (48%)		117,633 (63%)		172,109 (93%)	
Prescriptions/prescriber	25	6 [2, 20]	80	10 [3, 45]	34	9 [2, 40]
Patients/prescriber	6	3 [1, 7]	12	4 [2, 14]	27	7 [1, 29]
MME/prescription/patient	694	300 [145, 738]	709	311 [150, 713]	459	189 [110, 386]
MME/patient/prescriber	4152	567 [187, 5667]	4873	680 [200, 3124]	887	9228 [123, 580]
Patients/1000 MME/prescriber	3.8	1.8 [0.4, 5.3]	3.6	1.5 [0.3, 5.0]	6.1	4.4 [1.7, 8.1]

Prescribers can be in more than 1 category and are in a category if they wrote at least one prescription for a patient in that class.

MME = Morphine milligram equivalent

To understand the contribution of providers who had small prescription history with those patients like emergency, urgent care or other clinic providers, we employed two separate proxies likely to characterize their prescribing: the previously defined group of “episodic prescribers” and low frequency prescribers, defined as those who contributed 1 prescription to the patient [12]. Our data reflect that regardless of definition, both groups provided doctor shopping patients a small percentage of prescriptions (13.5% by the episodic and 15.0% by the 1 prescription definition) and an even smaller fraction of the total MME (1.7% and 4.9% respectively), despite having written at least one prescription for 88% (episodic) or 99% (1-prescription) of these patients. These prescribers were responsible for an even lower fraction of the opioids received by doctor shopping eligible patients: prescriptions 5.2% and 6.7%; MME 0.7% and 2.5%, respectively.

## Limitations

Prescriptions from patients using a variety of names, addresses, and dates of birth may fail to link to a single patient identifier, artificially increasing the number of individuals in the database and decreasing their average use. However, per California law, patients need to establish identity in order to obtain a controlled substance from a pharmacy, thus limiting the magnitude of this phenomenon [20]. The CURES database also does not include opioids that may have been purchased through clandestine routes or procured via other forms of diversion.

Our analysis also assumes that the majority of opioids obtained via prescription are consumed by the patient and not diverted. While some of those included in the doctor shopping group may be actively diverting opioids, it is difficult to identify these patients in the clinical setting and an overemphasis upon this contribution would pose a threat to the provider-patient relationship. This is especially true for episodic prescribers, who represent the majority of clinicians.

Within the CURES database were a small number of patient (.04%) and provider identifiers (.03%) associated with exorbitant amounts of opioids. We cannot tell if these are real or artefactual, for example, pharmacy errors, drug rehabilitation centers that obtained all medications under the same patient name, or teaching hospitals that used one physician identifier for all prescriptions written by not yet licensed house staff. We accounted for these rare outliers by using medians in addition to means. Lastly, individuals who use a clinic as their PCP may see different physicians at each visit and appear to be doctor shopping despite having a regular source of care. This would lead to an overestimation of the number of patients who doctor shop, a bias that would not affect our conclusions.

## Discussion

Our descriptive analysis of an 8-year sample of California’s PDMP provides several key insights into the prescription patterns of patients who doctor shop. First, those who doctor shop are relatively few in number (1.3% of all those receiving prescriptions). Second, they exhibit specific prescription acquisition patterns. They have the highest 90-day average daily MME use, putting them at higher risk of overdose [14–16]. They also demonstrate the longest active time in the database compared to other groups, and they have the highest frequency of addiction related opioid prescriptions and overall long acting opioid use—use that may be indicative of treatment for a chronic painful medical disorder, an opioid use disorder, or both. Third, while those who doctor shopped obtained prescriptions from a larger number of prescribers, the majority of their opioid prescriptions came from a small number of providers who consistently prescribed large amounts of opioids over long periods of time (similar to doctor shopping eligible patients). In comparison, episodic and low frequency prescribers of opioids prescribed less than 10% of the MME obtained by doctor shopping patients, even when they were doctor shopping (top of upper right panel in Fig 2). These findings contradict the assumption that those who doctor shop depend on multiple one-time emergency department or clinic visits as a main source of opioid prescriptions.

An unexpected finding regarding prescription patterns for doctor shopping versus doctor shopping eligible patients is that there is less difference than similarity between the two groups. When *not* doctor shopping, doctor shopping patients exhibit the same prescription profile as doctor shopping eligible patients, whereas when they *are* doctor shopping they rely less on their primary opioid suppliers and more on episodic providers. Presumably, the switch to doctor shopping could be triggered by an event, such as a destabilization in access. These time periods of doctor shopping, inductively, may represent a period of heightened vulnerability in which to intervene with referral to treatment [21].

From a policy standpoint, opioid prescription reduction among opioid naïve or low use patients is projected to have a modest effect, while prescription reduction from the episodic settings for higher risk patient groups like those patients who doctor shopped promises to be ineffective [22]. Over 70% of both doctor shopping and doctor shopping eligible patients exhibited high risk opioid use (≥ 20 MME for 90 days). To focus on the doctor shopping patient is to miss 7 of 8 patients who engage in high risk prescription opioid use [23]. Given the similarity in behavior between doctor shopping and doctor shopping eligible patients, the currently promoted usage of PDMPs to flag only those patients who doctor shop is ambiguous and may be harmful if used simply for the policing *purposes by providers*. This is true especially in light of evidence that doctor shopping is not associated with overdoses [24]. Rather, there should be a more nuanced approach to ensure PDMPs empower providers to address the underlying problem present in the patient who doctor shops. An example of this could be including a list of local opioid treatment centers in the PDMP report when patients are flagged.

Furthermore, even if all contributions from episodic and low frequency prescribers were removed, doctor shopping patients would still receive large amounts of MME from primary prescribers and have dangerous levels of opioid use over long periods of time. The more pragmatic move for healthcare systems and providers who are faced with a patient who doctor shops in their emergency department, clinic or urgent care center may be to provide conduits to treatment [21]. Such referral pathways to opioid use disorder treatment improve addiction treatment retention and patient mortality [25, 26]. This strategy will not fit all patients who doctor shop, but it will ensure that patients with high risk opioid use disorder are offered evidence-based treatment and that the therapeutic alliance is preserved. Providers across the country are piloting different forms of linkage to treatment for patients with opioid use disorder, especially with regards to medication assisted treatment [27–29]. Increasing treatment access and improving harm reduction programming represents a superior strategy for attenuating the opioid epidemic and should overshadow prescription reduction efforts in high risk populations.

## Supporting information

S1 Appendix (DOCX)

S2 Appendix (DOCX)
