# Supplementary material for: Opioid prescription patterns among patients who doctor shop; Implications for providers
Source: PLoS One. 2020 May 26;15(5):e0232533. doi: 10.1371/journal.pone.0232533 (PMC7250533; doi:10.1371/journal.pone.0232533)
Supplement: S1 Appendix — (DOCX) [file pone.0232533.s001.docx]

Supplementary Appendix 1:

For each patient we created the following variables as defined:

| Total time span | The number of days between the patient’s first and last prescription in the data base plus 90 days (to allow for the consumption of the final prescription) |
| --- | --- |
| Active time | The number of days that a patient was prescribed any amount of opioid medications. We calculated this as the number days in the database that are within 90-days of a prescription. For example, a patient who had 2 prescriptions in the database on Jan 1, 2008 and Dec 1, 2012, would have 180 days of active time, a patient who received 4 prescriptions on Jan 1, Jan 2, Jan 5, and Jan 7 of 2008 would have 97 days of active time. |
| MME (morphine milligram equivalents) per script | No. of pills multiplied by strength multiplied by conversion factor |
| MME/day | Total MME prescribed/active time |
| Maximum 90-day daily MME | We calculated total MME for each 90-day period during the patient’s time in database, selected the maximum value, and divided that total by 90. |
| Doctor shopping status | For each 183-day (6-month) period we determined the number or prescriptions and the number of unique providers who wrote those prescriptions. Patients were classified as “doctor shopping” if they had at least 6 different prescribers in any rolling 6-month period, “doctor shopping eligible” if they had 6 or more prescriptions but less than 6 providers in their maximum 6-month period, and “neither” if they met neither definition. |
| Episodic provider | A provider who writes: 95% of prescriptions for short acting opioids, 95% of prescriptions for ≤ 31 pills or ≤ 473 mls, 90% of prescriptions are for unique patients, ≤ 1% of prescriptions to patients who receive >5 prescriptions from that provider, < 540 prescriptions per year in the database[12]. |
| One-prescription provider prescription | A prescription written by a provider who wrote only 1 prescription for a patient during the 8-year database. |
| Two-prescription provider prescription | A prescription written by a provider who wrote exactly 2 prescriptions for a patient during the 8-year database. |
| Primary provider prescription | A prescription written by a provider who provided: a) >2 scripts to the patient, b) ≥ 30% of the patient’s prescriptions in the database c) ≥ 30% of remaining prescriptions after the highest % provider’s prescriptions are removed. Thus, there can be 2 primary providers so long as both wrote at least 3 scripts and one accounted for ≥ 30% of scripts and the other ≥ 30% of the remaining scripts. |
| Other provider prescription | A prescription written by a provider who does not meet the 1 or 2 prescription or primary provider definitions. |
