# Supplementary material for: Opioid prescription patterns among patients who doctor shop; Implications for providers
Source: PLoS One. 2020 May 26;15(5):e0232533. doi: 10.1371/journal.pone.0232533 (PMC7250533; doi:10.1371/journal.pone.0232533)
Supplement: S2 Appendix — (DOCX) [file pone.0232533.s002.docx]

Supplementary Appendix 2 – Medications prescribed by patient type

| Doctor shopping patient  (N= 2,198,631) | Doctor shopping eligible patient  (N= 9,378,221) | Neither  (N= 6,378,116) |
| --- | --- | --- |
| Hydrocodone – APAP 57% | Hydrocodone – APAP 59% | Hydrocodone – APAP 65% |
| Oxycodone – APAP 9% | Oxycodone – APAP 7% | Codeine – APAP 11% |
| Morphine sulfate 6% | Morphine sulfate 5% | Oxycodone – APAP 7% |
| Oxycodone 5% | Codeine – APAP 4% | Tramadol 4% |
| Hydromorphone 3% | Oxycodone 4% | Codeine – promethazine 2% |
| Codeine – APAP 3% | Methadone 3% | Propoxyphene – APAP 2% |
| Methadone 3% | Oxycontin 3% | Morphine sulfate 2% |
| Oxycontin 3% | Tramadol 2% | Codeine – guaifenesin 1% |
| Fentanyl 2% | Buprenorphine 2% | Oxycodone 1% |

APAP = acetaminophen
